# Exploring Protein-Based Fishmeal Alternatives for Aquaculture Feeds in Bangladesh

**DOI:** 10.1155/anu/3198303

**Published:** 2025-09-18

**Authors:** Md. Naim Mahmud, Farzana Yasmin Ritu, Abu Ayub Ansary, Mohammad Mahfujul Haque

**Affiliations:** Department of Aquaculture, Faculty of Fisheries, Bangladesh Agricultural University, Mymensingh, Bangladesh

**Keywords:** aquaculture, fish feed, fishmeal, protein sources, sustainability

## Abstract

The rapid expansion of aquaculture in Bangladesh has played a vital role in meeting the increasing demand for fish protein. However, the industry faces significant challenges due to the high cost and environmental impact of fishmeal (FM), a primary protein source in aquafeeds. This review critically evaluates alternative protein sources for aquafeeds, including plant-based proteins, insect meals, agricultural by-products, and single-cell protein (SCP), with a focus on their applicability in the Bangladesh context. Using the Joanna Briggs Institute (JBI) methodology, we synthesized evidence from peer-reviewed studies and institutional reports to assess the nutritional profiles of these alternatives. Results show that while plant-based proteins are affordable and locally available, they require processing to reduce antinutritional factors (ANFs). Insect meals exhibit high protein content and feed efficiency, with a primary focus on their essential amino acids (EAAs) profiles, which are crucial for optimal fish growth, immunity, and metabolic performance. Agricultural wastes such as fruit peels and vegetable residues offer cost-effective and immune-boosting properties, while SCP derived from algae, fungi, yeast, and bacteria emerge as a nutritionally robust and environmentally sustainable option. Despite the promise of these alternatives, limitations persist in terms of nutrient imbalances, processing requirements, and scalability. Overcoming these barriers demands targeted research and development, policy support, and investment in local feed innovation to ensure sustainable aquaculture growth. This study underscores the critical need for further research and strategic implementation of alternative feed resources to enhance the sustainability, profitability, and resilience of aquaculture in Bangladesh, with a focus on optimizing inclusion levels, improving digestibility, and utilizing locally available ingredients to ensure nutritional balance and food security.

## 1. Introduction

The rapid growth of the global human population has exerted tremendous pressure on the food production sector [1, 2]. Consequently, aquaculture has emerged as a crucial source of protein to meet the increasing demands of the growing population [3–5]. Since 1990, aquaculture production has increased by more than 650% [6–8]. In 2022, global aquaculture production reached a record high of 130.9 million MT, marking a 6.6% increase compared to 2020. Of this total, 94.4 million MT comprised aquatic animals, with Asia remaining the dominant contributor by accounting for 91.4% of global production [7]. Within this context, Bangladesh is a key player in global fish production, achieving a total output of 4.91 million MT in 2022–2023 [9]. Aquaculture has been instrumental in this success, accounting for 58.03% of the total fish production of the country [9]. Over the past three decades, aquaculture has emerged as the fastest-growing agro-food sector in Bangladesh. The inland aquaculture sector, in particular, has demonstrated a consistent upward trend, with production more than doubling from 1.006 million MT in 2007–08 to 2.852 million MT in 2022–23 [9, 10]. During the period of 2022–2023, carp species, including Indian major carps, indigenous carps, exotic carps, and catfish, contributed significantly to the total aquaculture production. The key species contributing to aquaculture production include tilapia/nilotica (*Oreochromis Mosambicus*/*O. Niloticus*), pangas (*Pangasius pangasius*), rohu (*Labeo rohita*), catla (*Catla catla*), mrigal (*Cirrhinus cirrhosus*), kalibaus (*L. calbasu*), bata (*L. bata*), gonia (*L. gonius*), silver carp (*Hypophthalmichthys molitrix*), grass carp (*Ctenopharyngodon idella*), common carp (*Cyprinus carpio*), and various other exotic carps. Among these species, the major contributors to national fish production are major carp (22.06%), Pangasius (8.21%), tilapia (8.57%), and shrimp/prawn (5.52%). The production of major carps, exotic carps, and other exotic fish has steadily increased from 2002 to 2022, with average growth rates of 5.05%, 5.26%, and 4.29%, respectively [11]. Among the major aquaculture crustacean species, *Penaeus monodon*, locally known as bagda, and *Macrobrachium rosenbergii*, known as golda, are the two primary export commodities of Bangladesh. These species are cultivated in various aquaculture systems, either intensive or semi-intensive, to meet the demand for farmed fish [9]. However, the sustainability of these systems is increasingly threatened by input-related challenges. Rising prices of commercial fish feed, particularly meat meal, fishmeal (FM), and soybean meal (SM), which make up 60%–70% of total production costs, have become a major challenge for intensive aquaculture systems and are limiting the sector's growth [3, 12]. FM is widely used as the primary and most expensive protein source in formulated fish diets. It is highly valued for its rich protein content, well-balanced essential amino acids (EAAs) profile, excellent nutrient digestibility, and low levels of antinutritional factors (ANFs) [4, 13]. Currently, approximately 90% of FM production comes from small pelagic fish species such as anchovies, sardines, mackerel, capelin, and menhaden [14]. However, ongoing concerns persist about the effects of fishing pressure on predator–prey dynamics within vulnerable marine ecosystems [15]. This situation necessitates the replacement of FM with more affordable alternative protein sources.

Adopting cost-effective and sustainable alternative protein sources can lower production costs, enhancing profitability and competitiveness in developing countries such as Bangladesh [16–18]. High feed costs impose a substantial economic burden on rural farmers, restricting their active participation in aquaculture and driving up market fish prices. While notable progress has been made in Bangladesh's aquaculture sector, the industry remains heavily reliant on imported feed ingredients, with feed expenditures accounting for approximately 70%–75% of total fish farming costs [19]. To enhance cost-efficiency while maintaining high nutritional quality, it is crucial to identify and incorporate low-cost alternative raw materials into feed formulations. Consequently, fish nutritionists and aquaculture researchers worldwide have increasingly focused on substituting FM with affordable and sustainable alternatives, such as plant protein ingredients (PPIs), insect-based proteins, agricultural by-products, single-cell protein (SCP), and other novel feedstuffs [4, 20–25]. Despite these promising options, research on alternative protein-based feed ingredients in Bangladesh remains limited, even though the country ranks fifth globally in aquaculture production [7]. Therefore, this study aims to evaluate conventional aquafeeds alongside the nutritional effectiveness and feasibility of alternative ingredients, such as plant proteins, insect-based proteins, agricultural by-products, and SCP as sustainable replacements for FM in Bangladesh's aquaculture industry. It also seeks to identify challenges and explore prospects for scaling these alternatives to support sustainable aquaculture practices.

## 2. Methodology

We applied the methodology outlined by the Joanna Briggs Institute (JBI) [26]. This approach was used to collect relevant data, including the defined inclusion and exclusion criteria, specifically focusing on the innovative fish feed alternatives for sustainable aquaculture in Bangladesh, following the search strategy [6]. Following conservative estimation norms, data on various fish culture methods, including production range, total production, and total cultured area, were obtained from the Fisheries Resources Survey System (FRSS) of the Department of Fisheries (DoF). Information on feed requirements per hectare for different culture methods was sourced from WorldFish reports [27]. The total feed requirement for each method was then calculated by multiplying the total fish farming area by the feed requirement per hectare, providing an estimate of the feed needed for fish production in Bangladesh.

### 2.1. Literature Search Strategy

A comprehensive literature search was conducted using databases such as PubMed, ScienceDirect, MedLine, Scopus, and Google Scholar to identify relevant literature. Different Boolean operators and keywords viz., “innovative fish feed,” “alternative fish feed ingredients,” “sustainable aquaculture,” “Bangladesh aquaculture,” “fish nutrition,” “feed formulation,” “plant-based feed,” “insect-based feed,” “single-cell protein,” “agricultural waste-based feed,” and “fish feed sustainability” are used to find out information on the websites.

### 2.2. Inclusion and Exclusion Criteria

To ensure relevance and quality, studies were selected based on the following criteria.

#### 2.2.1. Inclusion Criteria

Studies published within the last two decades (2005–present) to ensure relevance and recent advancements.

• Published in peer-reviewed journals, government reports, and organizational publications, all available in English.
• Focused on major aquaculture-producing countries worldwide, including both developed and developing nations, to ensure a comprehensive understanding of innovative fish feed alternatives and their global applicability.
• Studies that analyze the efficiency, cost-effectiveness, and sustainability of plant-based, insect-based, or other innovative feed ingredients.
• Clear outcomes related to feed ingredients used in aquaculture.

#### 2.2.2. Exclusion Criteria

• Documents not written in English.
• Duplicated studies are already included in other reviews.
• Research lacks empirical or analytical data.

### 2.3. Data Extraction

Data were systematically extracted from selected studies to evaluate innovative fish feed alternatives for sustainable aquaculture in Bangladesh. The extracted information included study identification details such as authors, publication year, journal, and title. The study design and methods were also analyzed, focusing on whether the study was experimental or observational, the species of aquaculture organisms examined, the type and source of alternative fish feed ingredients, and the methods used for feed formulation and application. Experimental details encompassed feed composition, inclusion levels of alternative ingredients, duration of feeding trials, and the control measures implemented to ensure accurate comparisons. Furthermore, key outcomes measured included growth rates, feed conversion ratios, survival rates, nutrient utilization, environmental impact, and cost-effectiveness of alternative feed ingredients. The results were synthesized by assessing both quantitative data, such as growth performance metrics and feed efficiency ratios, and qualitative findings, including observed behavioral responses, feed acceptance, and overall sustainability aspects of the alternative feed solutions.

### 2.4. Categorization and Synthesis

Extracted data were categorized based on the primary application areas of innovative protein-based fish feed alternatives in aquaculture, including plant-based proteins, insect-based feeds, microbial and algal-derived ingredients, and agro-industrial by-products. The synthesis process involved summarizing data to evaluate the overall effectiveness of various alternative feed ingredients for sustainable aquaculture in Bangladesh.

### 2.5. Critical Analysis and Interpretation

The critical analysis examined the effectiveness of innovative fish feed alternatives by assessing their nutritional value, cost-efficiency, and environmental impact. Findings were interpreted to identify the most sustainable options, highlighting benefits and challenges for aquaculture in Bangladesh.

## 3. Results and Discussion

### 3.1. Aquaculture Production and Estimated Total Amount of Feed Required

Inland aquaculture is the most prominent form of farming in Bangladesh, primarily due to the vast freshwater resources of the country. Notably, Bangladesh possesses a total of 846.3 thousand hectares dedicated to inland aquaculture [9]. Aquaculture methods in Bangladesh can be broadly categorized into four groups based on production intensity and input use: extensive, semi-intensive, intensive, and highly intensive [9, 11]. Understanding the distribution of these culture systems is essential for assessing input demand, particularly feed, which represents a major cost and sustainability challenge. As aquaculture continues to expand, improving feed efficiency and management will remain central to achieving sustainable growth. Inputs typically include external fish feed, stocking density, and chemical use, which increase progressively from extensive to highly intensive systems. Extensive and semi-intensive farms generally follow polyculture practices, whereas intensive and highly intensive systems predominantly rely on monoculture [28]. Extensive fish culture, with the lowest production rate (<1.5 MT/ha) (Table 1), contributes only 44,408 MT of total fish production across the area of 31,124 ha. This method has minimal feed requirements, estimated at 6412 MT, due to its reliance on natural productivity. In contrast, semi-intensive farming, which yields 1.5–4 MT/ha, significantly enhances production to 905,351 MT over 239,591 ha. This method requires 573,580 MT of feed, reflecting a moderate dependence on supplementary inputs.

Intensive and highly intensive fish culture methods demonstrate further production increases, but with substantial feed demands. Intensive culture, producing 4–10 MT/ha, contributes the highest total fish production (969,292 MT) from 126,306 ha, requiring 1,624,421 MT of feed. Highly intensive methods, with production exceeding 10 MT/ha, generate 353,616 MT from just 18,851 ha, yet have the highest feed requirement per hectare (62,063 kg), totaling 1,169,950 MT. Overall, the total fish production across all methods is 2,272,667 MT over 415,872 ha, with an estimated total feed demand of 3.37 million MT. These results emphasize the growing reliance on external feed as production intensifies, underscoring the need for efficient feed management and sustainable aquaculture practices. To enhance sustainable aquaculture practices, research and development efforts should focus on optimizing fish culture methods to improve productivity while minimizing feed dependency. Given the significant trade-offs between production intensity and feed requirements, priority should be given to developing efficient feed formulations, enhancing natural productivity in extensive and semi-intensive systems, and adopting advanced technologies for feed utilization in intensive and highly intensive farming.

### 3.2. Conventional Aquafeeds in Bangladesh

**Figure 1:**
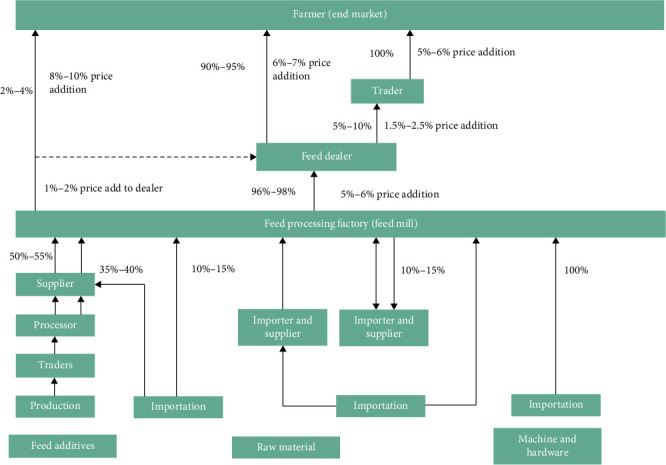
Feed value chain in Bangladesh (adapted from [19]).

Based on favorable temperatures and water availability, the main season for commercial fish culture in Bangladesh generally starts in March and continues until November, with the greatest volumes of aquafeeds being produced between April and September [29]. In 2020, nearly all farmers (95%) in Bangladesh used supplementary feeds in aquaculture [30]. Among them, 59% used both formulated and nonformulated feed types, while only 4% exclusively relied on formulated feed. Formulated feed accounted for 37% of the total feed usage, whereas nonformulated feed made up the remaining 63%. Notably, floating feed constituted 22% of the overall feed usage [30]. The supply chain of aquafeed in Bangladesh experiences a progressive increase in feed price at various stages from importation to the end market. Imported raw materials and additives contribute to a 10%–15% price increase at the feed processing factory level. Feed dealers further raise the price by 5%–6%, and traders add 1.5%–2.5% (Figure 1). By the time feed reaches farmers, these cumulative price additions result in a significant cost escalation. The average feed use rate in 2020 was 3.25 MT/ha, reflecting a 30%–38% increase since 2013 [27, 30]. The aquafeed sector in Bangladesh, despite its rapid growth, faces several limitations that require research and development interventions. One of the major challenges is the escalating cost of aquafeed driven by cumulative price increases along the supply chain, starting from imported raw materials to final retail prices. Dependency on imported ingredients makes the sector vulnerable to global price fluctuations, policy changes, and international trade dynamics. With the expansion of the aquaculture industry, the number of feed producers, importers, and retailers in Bangladesh has been rapidly increasing. Raw materials for aquafeed production are primarily supplied to feed mills through a network of distributors. The prices of the aquafeed ingredients fluctuate due to various factors, including global commodity price trends, changes in import policies, and foreign relations with exporting countries. To mitigate these fluctuations, locally available ingredients with high crude protein content are extensively used in aquafeed formulations across the country. Key components include mustard oil cake, broken rice, canola meal, blood meal, sorghum meal, copra meal, wheat bran, maize, and locally produced FM derived from trash fish, crabs, shrimp shells, dried fish offal from processing plants, and other aquatic animals [18, 29, 31]. Although locally available ingredients are increasingly used to mitigate these issues, their nutritional adequacy and consistency remain under-researched.

Additionally, while the use of supplementary feeds is widespread, there is still a high reliance on nonformulated feed, indicating gaps in farmers' access to affordable, high-quality formulated feeds. The growing demand for aquafeed, reflected in the rising feed use rates, further underscores the need for improved feed efficiency and cost-effective alternatives. Therefore, future research and development should focus on optimizing locally sourced feed formulations, enhancing feed processing technologies, improving the supply chain to reduce costs, and ensuring consistent feed quality to support sustainable aquaculture growth.

### 3.3. Alternative Fish Feed for Sustainable Aquaculture

In Bangladesh, fish play a crucial role in food and nutritional security, contributing approximately 60% of the daily animal protein intake (67.8 g/day). More than 98% of this comes from domestic production, highlighting the nation's strong reliance on aquaculture [9]. This rapid expansion has contributed to food security and economic development. However, despite the remarkable growth of aquaculture, this sector faces significant challenges, with high feed costs being one of the most critical constraints [30]. The sustainability of aquaculture depends on innovative and cost-effective feeding strategies that address ecological, economic, and ethical concerns [2]. Partial and complete FM replacement strategies are being adopted worldwide to make aquaculture more viable and sustainable [20]. The EAAs requirements of fish in dietary protein are critical for ensuring optimal growth, health, and metabolic function. The required levels are as lysine (5.18% ± 0.16%), methionine (3.04% ± 0.20%), threonine (3.22% ± 0.35%), arginine (4.66% ± 0.18%), histidine (1.88% ± 0.06%), valine (3.43% ± 0.09%), phenylalanine (5.16% ± 0.51%), isoleucine (2.91% ± 0.18%), leucine (4.20% ± 0.22%), and tryptophan (0.68% ± 0.06%) [32–34]. These amino acids must be supplied through feed, as fish cannot synthesize them endogenously. Their balanced inclusion is essential for efficient protein synthesis, immune responses, and overall physiological performance in aquaculture species.

To address the challenges of high feed costs, Bangladesh should prioritize the use of locally available ingredients rich in EAAs and explore alternative raw materials for sustainable fish feed formulation. Dependence on imported feed ingredients not only increases production costs but also raises concerns about environmental sustainability due to long-distance transportation. Novel ingredients and locally sourced aquafeeds may offer a viable solution to reduce costs and enhance self-sufficiency in the sector. Several alternative feed sources, including various grasses, insects, vegetable waste, aquatic weeds, plant leaves, seeds, SCP, and seed extracts, have been explored for fish feed formulations [16, 20, 35–38]. Among these, plant-based protein sources have gained particular attention due to their sustainability, environmental benefits, and cost-effectiveness [39]. Insects can be farmed on organic waste materials, promoting a circular economy and reducing the environmental impact of aquaculture operations [40] (Table 2). Whole insects offer a rich protein source, containing about 42%–63.3% crude protein on a dry matter basis, while defatted insect meal can contain up to 74% crude protein [3]. Additionally, fruit processing wastes and by-products present promising alternative feed options for aquaculture species, as they contain bioactive compounds and exogenous enzymes that improve nutritional value and enhance digestibility [52, 59]. SCP derived from various microbes also serves as a highly efficient protein source, containing 30%–70% protein, which is significantly higher than many plant and animal-based alternatives. Moreover, SCP is nutritionally superior due to its well-balanced amino acid profile, making it a more effective substitute for conventional protein sources in aquafeeds [60]. The integration of these alternative ingredients into aquafeeds can play a crucial role in addressing the challenges posed by rising FM prices while promoting sustainable and cost-effective aquaculture practices in Bangladesh.

#### 3.3.1. Plant-Based Feed Ingredients

Plant-based sources are considered a potential alternative for fish feed, offering a nutritional solution without compromising feed quality [22, 36]. These feeds are rich in proteins, a variety of amino acids, and fatty acids that are often absent in animal proteins [35]. The use of plant protein sources in the feed industry is also recognized as sustainable, environmentally friendly, and cost-effective [39], making them a viable substitute for conventional feed ingredients. However, despite these benefits, plant-based feeds have certain limitations, such as antinutritional elements and imbalances in essential nutrients [61]. To enhance their nutritional value, these plant materials undergo processing methods such as soaking, drying, and grinding, which help reduce the presence of ANFs before being added to fish diets [62]. Numerous studies have demonstrated that using plant ingredients or protein enhances fish productivity and overall health condition [63–65] (Table 3). Iskandar et al. [81] reported that incorporating 40% fermented *Lemna* spp. in artificial feed resulted in the highest growth of carp. Similarly, Mandal and Ghosh [82] found that the inclusion of 20% water lettuce in a low-cost, nonconventional diet for *L. rohita* led to better growth performance compared to the control diet. Additionally, Yousif et al. [75] observed that substituting 20% of FM with water spinach (*Ipomoea aquatica*) in the diet of Nile tilapia fry not only enhanced productivity but also reduced production costs. Beyond these specific examples, various plant-based ingredients, including grasses, vegetables, aquatic weeds, and their parts such as leaves, stems, seeds, and extracts, are commonly used in the fish feed industry [35]. Asian water grass, known for its soft texture, is a preferred diet of grass carp, while its roots are favored by tilapia (*O. niloticus*) and silver barb (*Puntius gonionotus*), contributing to lower fish production costs [83, 84]. Similarly, Refaey et al. [85] reported increased crude protein content in the whole body of *O. niloticus* when supplemented with 20% *Azolla* meal. These findings highlight the potential of plant-based feeds in promoting sustainable and cost-effective aquaculture practice.

Research and development initiatives are required to fully explore the potential of these plant-based sources as alternatives for fish feed in Bangladesh aquaculture. Although plant-based feeds are sustainable, cost-effective, and rich in essential nutrients. Therefore, further research is needed to identify locally available plant materials, improve processing techniques to enhance their nutritional value, and develop balanced formulations. Expanding such research will help ensure that plant-based feeds can effectively support fish growth, reduce production costs, and promote sustainable aquaculture practices in Bangladesh.

#### 3.3.2. Insect-Based Feed Ingredients

**Figure 2:**
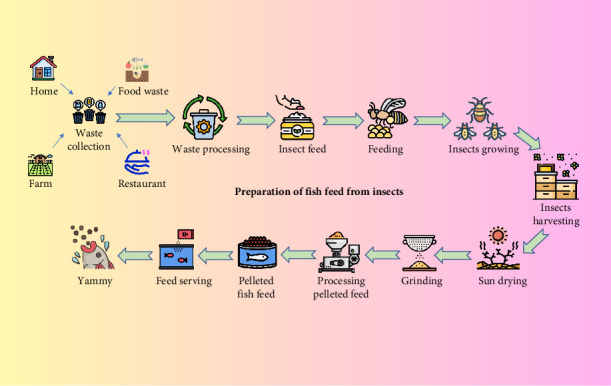
Conversion of food waste into insect-based fish feed for aquaculture (modified from [3]).

For the past two decades, research on insect meals as alternatives to FM and SM has been progressing steadily, with production rapidly increasing in regions such as China, Europe, North America, Australia, and Southeast Asia [86, 87]. Among the nearly one million recognized insect species worldwide, at least 16 have been evaluated as alternative protein sources for aquaculture [86, 87]. However, according to Alfiko et al. [3], only eight of these species have been scientifically reported to yield promising results. Black soldier fly (BSF) (*Hermetia illucens*) larvae contain a moderate amount of key EAAs, with lysine (3.60%), leucine (3.62%), valine (3.09%), and arginine (2.55%) being particularly notable. However, the methionine content (1.07%) is relatively low, which may limit its suitability as a sole protein source unless supplemented [88]. In contrast, mealworms (*Tenebrio molitor*) demonstrate a richer EAAs profile, especially in arginine (3.60%), alanine (4.53%), and glutamic acid (6.44%), along with reasonable levels of leucine (2.96%), isoleucine (4.12%), and methionine (1.76%) [89]. These values suggest that mealworms offer a more balanced protein source. Crickets (*Gryllus bimaculatus*) also exhibit strong concentrations of EAAs, including leucine (3.80%), lysine (3.22%), valine (4.50%), and arginine (3.92%), indicating their potential as a high-quality feed ingredient [90]. Likewise, superworms (*Zophobas morio*) present a rich and diverse EAAs composition, with leucine ranging from 3.4% to 4.5%, lysine from 2.4% to 2.9%, histidine from 1.4% to 2.3%, and arginine from 2.2% to 3.5%. However, as with other insect meals, methionine (0.5%–1.0%) and tryptophan (0.4%–0.5%) levels remain relatively low, highlighting the need for amino acid supplementation in feed formulations [91]. Studies indicate that insects can efficiently convert organic materials, such as food waste and manure, into valuable nutrient sources for livestock and aquaculture feed. This approach offers dual benefits by reducing waste and promoting environmental sustainability through nutrient recycling [92, 93] (Figure 2). The complete substitution of conventional feed ingredients in the diets of various aquaculture species has yielded promising results, particularly due to the high protein content of alternative ingredients [3, 94, 95] (Table 4). Insects not only provide high-quality protein but also contain EAAs, and are an excellent source of fatty acids and minerals [3, 97]. Also, they do not contain ANFs that can hamper feed utilization when fed to animals [101, 102].

Insects grow and reproduce rapidly, exhibit high feed conversion efficiency, and can be reared on biowaste, making them a promising component of sustainable aquaculture [103, 104]. In natural ecosystems, many fish species consume insects as part of their diet: omnivorous fish feed on insects at the bottom of water bodies, while juvenile carnivorous fish often begin life feeding on insects before transitioning to a fish-based diet [105]. Insects are capable of converting organic matter into high-quality protein, fat, and energy by efficiently assimilating and integrating amino acids and fatty acids from decomposing substrates into their biomass, thereby enhancing their nutritional value as feed for fish [106]. Numerous insect meals have been reported for use as supplementary feeds in fish culture, incorporated into the diet for preparing inexpensive aquafeed formulations (Table 5). Silkworm pupae meal seems to be a good protein source to replace part of FM in aquafeeds [122], applied in mirror carp (*C. carpio*) [123], rainbow shark (*Epalzeorhynchos frenatum*) [112], with benefits in terms of improving growth performance and particular physiological parameters. BSF larvae can be included in trout diets at up to 40% without negatively impacting growth, survival rate, condition factor, somatic indexes, fillet quality parameters, or intestinal morphology [124]. Another feeding experiment showed that replacing FM with defatted mealworm meal up to 80% in diets did not impact food intake or central homeostatic regulation, suggesting that defatted mealworm meal can serve as an FM substitute in European sea bass diets [125]. Additionally, mealworm meal may partially replace FM (up to 50%) in the feeds of rainbow trout [126].

There is a strong potential for research and development for using insect-based feed ingredients in Bangladesh aquaculture as sustainable alternatives to FM and SM. Insects such as BSFL, silkworm pupae, and mealworms are rich in high-quality protein, EAAs, fatty acids, and minerals, and they lack ANFs, making them suitable for fish diets. Globally, insects have been successfully used to replace a significant portion of FM in aquafeeds without compromising fish growth and health. Moreover, insects can be reared on organic waste, supporting nutrient recycling and reducing environmental pollution. Given their rapid growth, high feed conversion efficiency, and natural role in fish diets, further research in Bangladesh is needed to identify suitable insect species, optimize production methods, and develop cost-effective insect-based feeds to enhance sustainable aquaculture.

#### 3.3.3. Agricultural Waste-Based Feed Ingredients

Modern agricultural technologies have significantly increased global food production; however, they have also contributed to high levels of waste generation [127, 128]. Agricultural waste, which includes residues from plantation crops, livestock, fruit, and vegetable farming, poses environmental challenges when not managed properly [24]. Improper disposal can disrupt soil biological metabolisms, alter nutrient cycles, and generate greenhouse gases such as N_2_O, SO_2_, and CH_4_ [129]. Globally, an estimated 1.3 billion MT of food is wasted annually, accounting for 30% of all food produced [130]. This wastage has severe food security implications, as the lost food could feed over four times the 800 million people suffering from hunger [131]. In response to this issue, the United States Environmental Protection Agency (USEPA) introduced the food recovery hierarchy, which ranks waste management strategies by priority. Among these strategies, converting food waste into animal feed is considered one of the most effective and sustainable options. Agricultural waste is rich in nutrients but is not directly suitable for human consumption. Over the past two decades, researchers and animal feed manufacturers have explored its use in aquafeed formulations as a cost-effective alternative to traditional fish feed ingredients [132]. Vegetable waste, for instance, is a valuable raw material for fish feed due to its high protein content, and it is widely available, inexpensive, and does not compete with human dietary needs. Incorporating vegetable waste into aquafeeds not only reduces environmental pollution but also enhances sustainability in aquaculture [24]. Moreover, fruit processing by-products, such as banana peels, citrus fruit waste, and papaya waste, have been identified as promising feed ingredients due to their high content of bioactive compounds and exogenous enzymes [52, 59]. Banana peels, for example, are rich in potassium, polyphenols, flavonoids, catecholamines, fiber, and EAAs [51]. Interestingly, they contain higher polyphenol content than the fruit's flesh, offering antibacterial, antioxidative, and neuroprotective benefits [133, 134]. Studies have shown that incorporating banana peel flour into fish feed at a 5% inclusion level improves the health of rohu (*L. rohita*) [135]. Additionally, a 15% inclusion in common carp (*C. carpio*) for 8 weeks significantly enhanced growth performance, feed utilization, and nutrient retention [136]. Similarly, a 30% dietary inclusion in Nile tilapia (*O. niloticus*) improved growth performance, feed utilization, hepatosomatic index (HSI), and gonadosomatic index (GSI), although it slightly reduced the apparent digestibility coefficients (ADCs) of dry matter, CP, total carbohydrate, and gross energy [137]. Furthermore, African catfish (*C. gariepinus*) fed a 10% banana peel diet for 10 weeks exhibited improved FCR and PER [138]. In addition to banana peels, citrus fruit waste, including lemon and orange peels, has shown significant potential in fish feed. These by-products are rich in polyphenols, flavonoids, limonin, and triterpenoids, which contribute to improved fish health [139]. Fermented lemon peel, when included in the diet for 8 weeks, was found to enhance microvilli structure and lysozyme activity in Asian sea bass (*L. calcarifer*) [140]. Similarly, dried lemon peel inclusion at 2.5–5 g/kg for 60 days significantly increased growth performance, white blood cell count, and total protein content in *L. rohita* [141]. Likewise, orange peels, when incorporated at 2 g/kg for 60 days in Nile tilapia, resulted in significant growth improvement, increased intestinal villi height, and reduced FCR, thereby indicating better feed efficiency [142]. Additionally, Salem et al. [143] reported that supplementing the diet of gilthead seabream (*S. aurata*) with orange peel at concentrations ranging from 2.9 to 5.5 ppm of body weight for 60 consecutive days contributed to maintaining overall fish health. Besides fruit waste, other agricultural by-products such as yam peels, pineapple waste, and papaya waste have also been evaluated for their potential use in aquafeeds [144–146]. Yam peels contain bioactive compounds known as yam polysaccharides, which promote beneficial microbial growth while inhibiting pathogenic bacteria like *Vibrio* and *Pseudomonas* [145]. Pineapple waste, including the crown, skin, and core, is rich in bromelain, a proteolytic enzyme that enhances digestion and supports immune function in tilapia [147]. Similarly, papaya waste contains papain, another proteolytic enzyme unique to *Carica papaya*, which has demonstrated positive effects on fish growth and health, and various studies have shown that extracts from papaya leaves, skin, and seeds significantly improve growth performance and blood parameters in multiple fish species [24, 148]. For instance, feeding common carp fingerlings a diet containing 2.5% papaya leaf led to enhanced growth performance [144]. Furthermore, vegetable waste powder, derived from a mix of dried potato peels, cabbage, carrot peels, beetroot, and cauliflower, has also shown promising results in aquafeed. Rebecca and Bhavan [149] found that incorporating 15% VWP into the diet of *M. rosenbergii* postlarvae significantly enhanced weight gain, survival rates, and protein utilization. These findings suggest that incorporating agricultural waste in aquafeed not only provides a sustainable solution for food waste management but also enhances fish health and productivity. In the future, the utilization of agricultural waste as an alternative protein source in aquafeeds could become a sustainable and cost-effective solution for addressing food waste in Bangladesh while enhancing fish growth, immune function, and feed efficiency. By-products such as banana peels, citrus peels, yam peels, pineapple waste, papaya waste, and vegetable waste have the potential to provide bioactive compounds, proteolytic enzymes, and essential nutrients that support fish health. As the demand for sustainable aquaculture is expected to rise in Bangladesh, incorporating fruit and vegetable by-products into fish feed could play a crucial role in minimizing environmental pollution, reducing feed costs, and improving resource efficiency in aquaculture.

#### 3.3.4. SCP-Based Feed Ingredients

**Figure 3:**
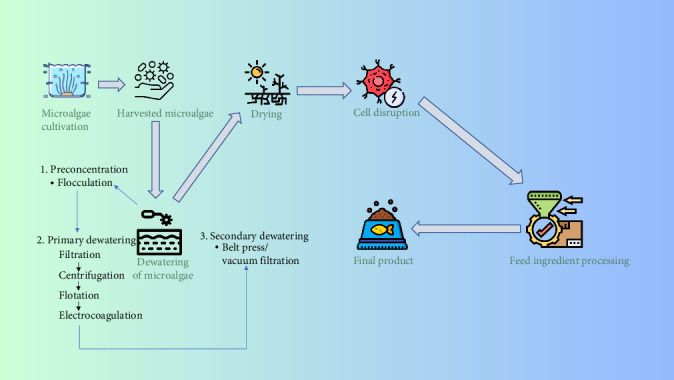
Preparation of fish feed from microalgae (modified from [174]).

SCP is considered the most appropriate as it is produced from single-celled organisms. SCP is derived from various microbial species, including algae, bacteria, fungi, and yeast, and has the highest potential as an alternative aquaculture feed ingredient [150, 151]. SCP derived from different microbes contains a high protein content (30%–70%), surpassing many green plants and animal protein sources [60, 152, 153] (Table 6). Furthermore, SCP proteins possess an excellent amino acid profile, making them nutritionally superior to conventional protein sources [60]. It derived from bacterial and algal sources, offers a well-balanced EAAs profile, positioning it as a promising alternative protein source for aquaculture feeds. Bacterial SCP, for example, contains substantial levels of key amino acids such as arginine (3.67%), lysine (4.30%), leucine (5.43%), valine (4.16%), and isoleucine (3.34%), which together form a significant part of its nutritional composition. Likewise, algal SCP, particularly *Spirulina maxima*, exhibits a similarly favorable profile, with mean concentrations of arginine (4.09%), lysine (2.87%), leucine (5.12%), valine (3.84%), and isoleucine (3.72%) [165]. The presence of these EAAs in balanced proportions suggests that SCP can effectively meet the dietary requirements of farmed fish species. Moreover, SCP contains various essential vitamins, including riboflavin, thiamine, pyridoxine, niacin, choline, folic acid, pantothenic acid, biotin, para-aminobenzoic acid, inositol, and vitamin B12 [166, 167]. SCP is produced when microorganisms utilize available waste materials as growth media, increasing their cell mass, which consists of SCP [168]. Several bacterial species have long been used in animal feed production due to their ability to generate SCP in large quantities. These include *Methylophilus methylotrophus*, *Bacillus megaterium*, *Acinetobacter calcoaceticus*, *Aeromonas hydrophila*, *Cellulomonas* spp., *Bacillus subtilis*, *Methylomonas methylotrophus*, *Achromobacter delvaevate*, *Thermomonospora fusca*, *Lactobacillus* spp., *Rhodopseudomonas capsulata*, *Flavobacterium* spp., and *Pseudomonas fluorescens* [169]. Recent studies have shown promising results when methanotrophic bacteria are used as a fish meal replacement, leading to increased growth efficiency, improved FCR, and enhanced gut health in Atlantic salmon and rainbow trout. Various fungal species also contribute to SCP production. Fungal SCP is typically rich in lysine and threonine, but deficient in cysteine and methionine, which are sulfur-containing amino acids mainly derived from plant sources [170]. Yeast-based SCP typically contains 55%–60% protein and around 15% nucleic acids on a dry weight basis [152]. Yeast-based SCP also serves as a potential protein replacement in animal feed, providing antioxidant and immunomodulatory effects [166]. A commercial example is Novacq, a potent microbial bioactive that reduces the quantity of fish meal required in black tiger prawn (*P. monodon*) feed while maintaining growth rates [171, 172]. Microalgae are widely cultivated and used as a feed resource in aquaculture, particularly during the larval rearing stage of many species [173]. Though water makes up nearly 99.8%–99.9% of microalgae-biomass [164], and microfiltration and centrifuge technologies are commonly used in such dewatering infrastructure [174] (Figure 3), dried algal meal can be used directly in fish feed, especially if the algae cell wall is fractured to maximize nutrient accessibility [175].

The nutritional quality of microalgae is exceptionally high, with a crude protein content of up to 71% and lipid content of up to 40% (Table 6), comparable to terrestrial plant and animal sources [176, 177]. Microalgae contain polyunsaturated fatty acids (PUFAs), such as docosahexaenoic acid (DHA), eicosapentaenoic acid (EPA), alpha-linolenic acid (ALA), and arachidonic acid (AA). These essential fatty acids are critical for fish development. Additionally, microalgal pigments, growth-promoting compounds, and hormones exhibit antioxidant, antibacterial, anti-inflammatory, and immune-stimulant properties, benefiting both marine and freshwater species [178, 179]. Numerous studies have demonstrated the successful use of microalgal biomass as a feed additive or FM replacement for a variety of aquaculture species, generally yielding positive effects on growth and quality [180–185] (Table 7).

Sherif et al. [205] reported that supplementing tilapia feed with 5–10 g *Spirulina* (*A. platensis*) per kg of feed helped reduce mortality rates and improve immunity in fish exposed to lead nitrate contamination. Basri et al. [206] replaced FM with microalgal biomass in the diet of juvenile Pacific white shrimp (*L. vannamei*), observing positive effects on growth performance. Gong et al. [207] found that intensely compressed microalgal biomass improved the digestibility of Atlantic salmon. The optimal inclusion level of microalgal meal in aquafeed varies depending on the microalgal species and aquaculture species being targeted.

In Bangladesh, SCP derived from bacteria, fungi, yeast, and microalgae presents a promising solution for enhancing aquaculture sustainability by providing a high-protein, nutrient-rich alternative to conventional feed ingredients. With its abundant EAAs and bioactive compounds, SCP can significantly improve fish growth, immunity, and nutrient absorption, which is crucial for the country's expanding aquaculture sector. Moreover, the ability of SCP-producing microorganisms to utilize agricultural and industrial waste materials aligns with Bangladesh's efforts to reduce environmental pollution and promote resource efficiency. As research and development in sustainable aquafeed advance, optimizing SCP and microalgae-based diets will be essential in reducing dependence on FM and SM, ensuring cost-effective, eco-friendly, and resilient aquaculture practices to support food security and livelihoods.

Although SCP is not yet utilized at the grassroots level in Bangladesh, research and development on the use of SCP in aquaculture holds significant potential for enhancing sustainability and reducing reliance on traditional feed ingredients like FM and SM. SCP, derived from bacteria, fungi, yeast, and microalgae, offers a high-protein, nutrient-rich alternative, with EAAs and bioactive compounds that can improve fish growth, immunity, and nutrient absorption. Moreover, SCP production from agricultural and industrial waste aligns with Bangladesh's goals for environmental sustainability and resource efficiency. As research progresses, optimizing SCP-based diets could provide a cost-effective, eco-friendly solution to support the growth of the aquaculture sector of Bangladesh while ensuring food security and economic resilience.

### 3.4. Challenges in Utilizing Alternative Feeds for Sustainable Aquaculture

Plant-based protein sources have been widely explored as alternatives to FM in aquafeed due to their availability and cost-effectiveness. However, they present significant challenges, including imbalanced amino acid profiles, low protein content, and the presence of ANFs that reduce nutrient absorption and affect fish health [208]. Additionally, plant-derived feeds often lack essential PUFAs and micronutrients critical for optimal fish growth, particularly in larval and nursery-stage fish [209]. One of the key challenges in using plant-based agricultural waste in aquafeeds is the presence of ANFs such as tannins, phytates, oxalates, saponins, lectins, alkaloids, protease inhibitors, and cyanogenic glycosides [210]. While some ANFs can provide beneficial effects at low concentrations, excessive amounts can negatively affect fish health and growth. Various techniques, including soaking, germination, boiling, autoclaving, fermentation, and genetic manipulation, have been employed to reduce ANFs content without significantly affecting the nutritional profile of plant-based proteins [211]. Additionally, incorporating enzymes or bacteria into plant-based feeds can help improve digestibility by breaking down ANFs in the fish intestine [36]. Insect meals have emerged as promising alternatives to FM due to their high protein content and ability to convert organic waste into biomass. However, certain challenges must be addressed before large-scale adoption in aquaculture. One major limitation is the relatively low levels of PUFAs, making insect meals less suitable for marine fish species that require high omega-3 fatty acid content [212]. Additionally, the exoskeletons of insects contain chitin, an indigestible polysaccharide that can affect protein digestibility despite the presence of chitinase enzymes in some fish species [213]. The bioaccumulation of pesticides and heavy metals in insects fed on organic waste also raises food safety concerns [214]. The economic viability of insect-based aquafeeds is influenced by factors such as the production system, the type of substrate used, and the location of the production facility [215]. Although large-scale insect farming is still developing [212], research has demonstrated both positive and negative effects of insect meal in fish diets. For instance, replacing more than 75% of FM with defatted BSFL meal caused intestinal tissue damage in juvenile Jian carp, likely due to chitin content [216]. Additionally, mealworm larvae diets resulted in taurine deficiency and EAAs reductions in rainbow trout [126]. To improve the digestibility and nutritional value of insect meals, enzymatic or bacterial fermentation methods can be employed. Research has shown that fermentation with *Bacillus subtilis* can reduce crude fiber content in BSFL from 20% to 13%, making it more digestible for fish [217]. Despite these technological advances, consumer acceptance remains a challenge, as many people may be hesitant to consume fish raised on insect-based diets, particularly if waste-fed insects are used [218]. The bioconversion of agricultural and industrial food waste into aquafeed ingredients presents a potential pathway for achieving a circular economy. However, certain limitations need to be addressed before widespread implementation. One key concern is the bioaccumulation of heavy metals in waste-fed insects and earthworms, which could ultimately pose food safety risks in aquaculture [214]. Several studies have reported negative effects of earthworm meal in fish diets, raising concerns about economic and environmental impacts [218]. Recent research has suggested that incorporating waste streams into aquafeeds must be carefully monitored to avoid food safety risks. Further research is required to assess the economic feasibility and scalability of waste valorization processes for aquafeed production. SCP derived from microbial fermentation offers a sustainable and low-carbon alternative to conventional protein sources. Unlike traditional aquafeed ingredients, SCP does not require extensive land, freshwater, or marine resources, making it an attractive option for reducing the environmental footprint of aquaculture. However, SCP also presents several challenges, including high nucleic acid concentrations, which can lead to increased uric acid levels and potential kidney stone formation in fish [167]. Additionally, certain microorganisms used in SCP production can generate toxic substances, such as mycotoxins and cyanotoxins, necessitating careful strain selection and processing [167]. To improve SCP digestibility and safety, various physical and chemical treatments have been explored. For example, enzymatic processing can reduce nucleic acid content, improving SCP suitability as an aquafeed ingredient [196]. SCP aligns well with circular bioeconomy principles, as it can utilize organic waste streams as substrates for microbial growth, reducing overall environmental impact [219]. Future research should focus on optimizing SCP production methods to enhance cost-efficiency and commercial viability. Microalgae, a type of SCP, have been explored as an alternative ingredient in aquafeeds due to their high protein and essential nutrient content. However, some species contain ANFs that can reduce enzyme activity in fish, leading to impaired digestion and nutrient absorption. For example, species such as *Tetradesmus obliquus*, *Kirchneriella lunaris*, and *Pseudokirchneriella subcapitata* have been shown to negatively impact liver enzyme activity in Japanese seabass (*Lateolabrax japonicus*) [220]. Methods such as solid-state fermentation using *Aspergillus sojae* and *Aspergillus ficuum* have been successful in reducing phytic acid content, thereby improving nutrient availability [221]. Additionally, microalgae can play a role in mitigating environmental concerns related to heavy metal contamination. For instance, *Spirulina* supplementation has been shown to reduce metal toxicity in fish [222]. Due to the presence of nonstarch components and tough cell walls, different microalgae species have low digestibility, necessitating various treatment methods such as enzymatic hydrolysis and physical processing [207, 223, 224]. Future research should focus on optimizing microalgae production and processing for aquafeeds to maximize their benefits. The development of alternative protein sources is crucial for ensuring the long-term sustainability of aquaculture in Bangladesh. Given the country's growing aquaculture sector and dependence on FM, exploring plant-based proteins, insect meals, agricultural waste bioconversion, and SCP, including microalgae, presents promising opportunities. However, each alternative faces specific challenges. Plant proteins require strategies to reduce ANFs to enhance digestibility, while insect meals need further research to improve nutrient assimilation and consumer acceptance. Agricultural waste valorization must address food safety and regulatory concerns, and SCP must overcome limitations related to high nucleic acid content. In Bangladesh, extensive research has been conducted on aquaculture covering small-scale and commercial practices as well as the impacts of climate change [225–228] but future research should focus on optimizing these protein sources through advancements in biotechnology, feed formulation, and policy support to achieve an environmentally sustainable and economically viable aquaculture industry.

## 4. Conclusion and Future Directions

Aquaculture has become a cornerstone of Bangladesh's food security and economic development. However, the sector faces critical challenges due to its heavy reliance on FM, which is both costly and environmentally unsustainable. This study highlights the potential of protein-based alternative feed ingredients, such as plant-based proteins, insect meals, agricultural by-products, and SCP, as viable replacements for FM in aquafeeds. These alternatives have demonstrated promising results in improving fish growth, FCR, PER, immune response, and overall sustainability. Despite their advantages, issues related to digestibility, ANFs, production scalability, and consumer acceptance must be addressed before they can be widely adopted.

To ensure the long-term sustainability of aquaculture in Bangladesh, future research should focus on optimizing feed formulations by combining multiple alternative protein sources to achieve a nutritionally balanced diet with improved digestibility and growth performance. Biotechnological advancements, such as fermentation, enzymatic treatment, and genetic modifications, could further enhance the nutritional value of plant-based and SCPs, making them more effective substitutes for FM. Additionally, large-scale production of insect-based feeds and microalgae should be explored using cost-effective farming techniques to ensure economic viability. The utilization of agro-industrial by-products, such as fruit peels and vegetable waste, also presents an opportunity for developing low-cost, sustainable feed ingredients that can reduce dependency on imported feed components. For widespread adoption of alternative fish feed ingredients in Bangladesh, a multistakeholder approach is essential, involving strong policy interventions, financial incentives, research institutions, and farmer engagement. Government policies should focus on providing subsidies and financial assistance to encourage the production and utilization of alternative feed ingredients while ensuring quality assurance and regulatory frameworks that promote their safe and effective use. Establishing certification and labeling systems for sustainable feed ingredients could further enhance market acceptance and consumer confidence.

In addition to policy support, academic and research institutions, particularly universities, play a crucial role in advancing sustainable aquafeed solutions. Universities should expand their research on feed formulation, ingredient processing, and nutritional efficacy, ensuring that alternative feed ingredients meet the dietary requirements of various aquaculture species. Collaborative projects between universities, government agencies, and the private sector can facilitate innovation in biotechnology, such as fermentation and enzymatic treatments, to improve the digestibility and bioavailability of plant and microbe-based proteins. Moreover, universities can contribute by developing pilot-scale production systems for insect meal and microalgae cultivation, creating scalable models that farmers and feed industries can adopt.

Beyond research, universities and extension services should focus on capacity-building initiatives for farmers and aquafeed producers. Conducting training programs, workshops, and field demonstrations on the benefits, formulation techniques, and proper utilization of alternative feeds will be critical in ensuring widespread adoption. Universities can also develop farmer advisory services and digital platforms that provide real-time guidance on cost-effective and nutritionally balanced feed options.

Furthermore, conducting environmental and economic assessments through life cycle assessment (LCA) studies is essential to evaluate the sustainability of alternative feed ingredients. Universities and research institutions should take the lead in these assessments, ensuring that the transition to alternative feeds contributes to reducing carbon footprints and enhancing resource efficiency in aquaculture. By integrating these strategies, like policy support, university-driven research and innovation, farmer training, and sustainability assessments in Bangladesh can build a more resilient, cost-effective, and environmentally sustainable aquaculture industry, reducing dependence on FM while strengthening food security and economic growth.

## Figures and Tables

**Table 1:** Estimated feed requirements across different aquaculture methods in Bangladesh.

Culture methods	Production range (MT/ha)	Total production (MT)	Total area (ha)	Required feed (kg/ha)	Estimated total feed (kg)	Estimated total feed (MT)
Extensive	<1.5	44,408	31,124	206	6,411,544	6412
Semi-intensive	1.5–4	905,351	239,591	2394	573,580,854	573,580
Intensive	>4–10	969,292	126,306	12,861	1,624,421,466	1,624,421
Highly intensive	>10	353,616	18,851	62,063	1,169,949,613	1,169,950

Total	—	2,272,667	415,872	77,524	3,374,363,477	33,74,363

**Table 2:** Comparative analysis of the studied fish feed ingredients.

Aquafeed	Availability	Potential benefits	References
Plant-based	High	• Readily available ingredients. • Sustainable fishing reduces pressure on fish stocks. • Safer for human consumption. • Cost-effective. • Contain essential and nonessential amino acids.	[24, 41–46]
Insect-based	Moderate	• High protein content. • Larvae contain natural antibiotics. • Sustainable, uses organic waste. • Good source of vitamins and minerals. • Contain beneficial unsaturated fatty acids. • High feed conversion efficiency.	[3, 40, 47–50]
Agricultural waste-based	High	• Contain antibacterial, antioxidative, and neuroprotective properties. • Waste utilization and environmental sustainability. • Budget-friendly alternative to conventional fish feed. • Contain natural probiotics that help boost the immune system.	[24, 51–53]
Single-cell protein-based	Low	• Highly nutritious. • Have a very fast growth rate that yields a high amount of production. • Require minimal water and arable land. • Improves growth rates and overall animal health. • Rich source of protein, vitamins, enzymes, minerals, and well-balanced amino acids.	[54–58]

**Table 3:** Effects of plant-based diets on fish and shellfish.

Plants	Fish species	Inclusion (%)	Culture period	CP (%)	Initial B. W (g)	Final B. W (g)	Effect	References
Moringa leaf (*M. oleifera*)	Mrigal carp (*C. mrigala*)	0, 10, 20,30, 40,50	90 days	30	6.35	20.63	Optimal growth and nutrient digestibility at 10% inclusion.	Tabassum et al. [66]
Moringa leaf (*M. oleifera*)	Nile tilapia (*O. niloticus*)	0, 10, 20	60 days	—	29.06	56.14	FCR and SGR were found to be the highest at 20%.	Nadia et al. [67]
Moringa leaf (*M. oleifera*)	Silver barb (*P. gonionotus*)	0, 10, 30, 50	60 days	30	5.70	20.85	50% inclusion exhibited the highest stress tolerance, with RBC, WBC, and hemoglobin increasing gradually.	Farhad et al. [68]
Moringa leaf (*M. oleifera*)	Gibel carp (*Carassius auratus gibelio*)	20, 40, 60	50 days	35	19.35	39.51	Enhanced growth, antioxidant and immune response, and resistance to *A. hydrophila* in a 40% inclusion level.	Zhang et al. [69]
Moringa leaf (*M. oleifera*)	Common carp (*C. carpio*)	0, 5, 10, 15	56 days	—	8.55	16.59	The best feed conversion rate and better growth were observed in fish fed with 5% moringa leaf meal.	Al-Dubakel and Taher [70]
Alfalfa leaf (*Medicago sativa*)	Goldfish (*C. auratus*)	0, 5, 10, 15, 25, 40	60 days	40–41	10.24	23.70	A 15% dietary inclusion of alfalfa is suitable for ensuring good pigmentation, acceptable growth, and efficient feed utilization.	Yanar et al. [71]
Cassava leaf (*Manihot esculenta*)	Nile tilapia (*O. niloticus*)	30	60 days	30	11.4–12.2	101.4	Diets with 30% fermented cassava leaves had overall acceptable growth performance with better production outcomes for Nile tilapia farming.	Amare et al. [37]
Subabul leaf (*Leucaena leucocephala*)	African catfish (*C. gariepinus*)	0, 5, 10, 15, 20	8 weeks	35	7.50	15.16	Fish-fed 20% meal had the best performance in terms of weight gain, SGR, and FCR.	Tiamiyu et al. [72]
Duckweed (*Lemna minor*)	Nile tilapia (*O*. *niloticus*)	25, 50, 75, 100	56 days	30	4.10	28.70	25% inclusion showed the best growth performance and feed utilization.	Ibrahim et al. [73]
Duckweed (*L. minor*)	Common carp (*C. carpio*)	15, 30, 45	60 days	32	16.25	23.01	Up to 15% inclusion demonstrated better growth performance.	Asimi et al. [74]
Water spinach (*Ipomoea* *aquatica*)	Nile tilapia (*O*. *niloticus*)	25, 50, 75, 100	7 weeks	30	3.60	40.4	25% inclusion showed better growth performance and feed efficiency.	Yousif et al. [75]
Water hyacinth (*Eichhornia* *crassipes*)	Common carp (*C. carpio*)	10, 20, 30, 40	70 days	35–38	1.20	5.06	40% replacement showed better growth performance and cost-effectiveness.	Mohapatra [76]
Aquatic fern (*Azolla pinnata*)	Thai silver barb (*Barbonymus* *gonionotus*)	25, 50, 75, 100	56 days	35	3.90	30.93	25% fresh Azolla inclusion resulted in better growth performance, a higher protein efficiency ratio (PER), and cost-effectiveness.	Das et al. [77]
Water lettuce (*Pistia tratiotes*)	Rohu (*L. rohita*)	10, 20, 30, 40, 50	80 days	25–35	1.23	2.75	30% showed better growth performance and FCR.	Nisha and Geetha [78]
Water lily (*Nymphaea* sp.)	Common carp (*C. carpio*)	30, 40, 50	45 days	27–32	1.50	36.7	40% showed the best growth performance and survival.	Sivani et al. [79]
Taro leaves (*Colocasia* *esculenta*)	Nile tilapia (*O*. *niloticus*)	33, 67, 100	84 days	23	73	160.5	33% showed better survival, growth performance, and cost-effectiveness.	Mathia and Fotedar [80]

Abbreviations: B.W, body weight; CP, crude protein.

**Table 4:** Main nutritional components (%) of commonly used insects in aquaculture.

Insect species	Protein (%)	Lipid (%)	Ash (%)	Fiber (%)	References
Superworm (*Z. morio*)	53.32	40	0.23	2.30	Wadje et al. [96]
Mealworm (*T. molitor*)	49.1	38.3	4.1	8.5	Liu et al. [97]
Black soldier fly (*H. illucens*)	48.20	25.69	8.27	9.96	Zulkifli et al. [88]
Cricket (*G. bimaculatus*)	54.10	26.90	2.2	6.90	Jayanegara et al. [98]
Silkworm (*Bombyx mori*)	57.21	31.29	4.01	2.39	Bhagat and Barat [99]
Housefly maggots (*Musca domestica*)	56.96	24.56	6.80	5.54	Obeng et al. [100]

**Table 5:** Use of insects as feed ingredients in different aquaculture species.

Insects	Fish species	Inclusion (%)	Culture period	CP (%)	Initial B. W (g)	Final B. W (g)	Effect	References
Housefly maggots (*M. domestica*)	Tilapia (*O. niloticus*)	66% dried and fresh	56 days	35	10.39	52.24	The dried form of the housefly maggot exhibited superior growth performance compared to the live form.	[107]
BSF (*H. illucens*)	African catfish (*C. gariepinus*)	0, 25, 50, 75, 100	12 weeks	40	0.46	14.37	BSFL meal can replace up to 25% of fishmeal without significantly affecting growth performance and feed utilization indices.	[108]
Cricket (*G. bimaculatus*)	Striped snakehead (*Channa striata*)	0, 50, 100	10 weeks	42.50	15	57	Growth performance and protein retention improved with higher inclusion levels of dietary cricket meal.	[109]
Cricket (*G. bimaculatus*)	Nile Tilapia (*O. niloticus*)	0, 25, 50, 75, 100	8 weeks	35.5	0.26	1.16	25% cricket meal inclusion improves feed efficiency.	[110]
Cricket (*G. bimaculatus*)	Largemouth bass (*Micropterus salmoides*)	0, 15, 30, 45, 60	8 weeks	53	19.87	56.51	There were significant differences in final body weight, SGR, FCR, viscerosomatic index, and survival rate among fish fed 15%, 30%, and 45% inclusion levels compared to the control diet.	[111]
Silkworm (*B. mori*)	Rainbow shark (*E. frenatum*)	30, 40, 50	8.5 weeks	35	1.44	6.62	A significant increase in SGR and PER was observed at 30% inclusion, while the FCR decreased.	[112]
Mealworm (*T. molitor*)	Rainbow trout (*O. mykiss*)	20, 30, 60, 100	90 days	48.5	5.1	55.9	100% replacement led to the highest weight gain and the most efficient FCR.	[94]
Mealworm (*T. molitor*)	Pacific white shrimp (*L. vannamei*)	0, 25, 50, 75, 100	8 weeks	35	1.5–1.6	5.27	The highest weight gain and optimal FCR were observed when 50% of fishmeal was replaced with mealworm.	[113]
Silkworm (*B. mori*)	Pacific white shrimp (*L. vannamei*)	25, 50, 75, 100	8 weeks	40	0.2	5.38	The complete replacement of fishmeal had no impact on shrimp growth but positively affected diet digestibility, antioxidant capacity, and molting time.	[114]
BSF (*H. illucens*)	Nile tilapia (*O. niloticus*)	0, 25, 50, 75, 100	56 days	30	3	11.83	The FCR and PER values at 50% inclusion were superior to those observed with other diets.	[115]
BSF (*H. illucens*)	Pacific white shrimp (*L. vannamei*)	7, 14, 21, 28, 36	63 days	35	1.24	15.95	Final weight, weight gain, SGR, and FCR were maintained when fishmeal replacement with BSFL meal was limited to less than 25% of the diet.	[116]
Mealworm (*T. molitor*)	Gilthead sea bream (*Sparus aurata*)	25, 50	163 days	43	105	294	Live weight gain, FCR, and PER were highest at 25% and 50% inclusion levels.	[117]
BSF (*H. illucens*)	Yellow catfish (*Peltobagrus fluvidraco*)	0, 10, 15, 20, 25, 30	8 weeks	42	1.17	20.7	Replacing 20% of fishmeal in the control diet with BSF did not significantly affect weight gain, FCR, and muscle proximate composition.	[118]
Silkworm (*B. mori*)	African catfish (*C. gariepinus*)	0, 25, 50, 75, 100	40 days	40	1.96	9	Fish growth rate and feed utilization parameters were higher in fingerlings fed diets containing 50% silkworm pupa.	[119]
Housefly maggots (*M. domestica*)	Red sea bream (*P. major*)	0.05, 0.5, 5	6 months	47–48	26.06	41.38	Red sea bream growth improved with increasing dietary housefly meal, with the lowest FCR observed at a 5% inclusion level.	[120]
Superworm (*Z. morio*)	Nile tilapia (*O. niloticus*)	0, 25, 50, 75, 100	8 weeks	32	5.57	10.24	Live weight gain, SGR, FCR, and PER were highest at 25% and 50% fishmeal replacement levels.	[121]

Abbreviations: B.W, body weight; CP, crude protein.

**Table 6:** Nutritional composition of various microalgae (DM basis).

Microalgae species	Protein (%)	Lipid (%)	Carbohydrate (%)	References
*Arthrospira platensis*	57–65	7–23	20–30	[153]
*Acutodesmus obliquus*	40.6	15.3	17	[154]
*Botryococcus brauni*	40	34	19	[155]
*Chlamydomonas*	47–50	20–25	18–20	[156]
*Chlorella vulgaris*	30–35	10–15	30–45	[157]
*C. sorokiniana*	50–55	20–25	15–18	[158]
*Dunaliella* spp.	50–70	25–35	10–25	[159]
*D. bioculata*	45–50	8–15	5–10	[160]
*Euglena gracilis*	45–55	25–35	15–20	[161]
*Haematococcus pluvialis*	48	15	27	[162]
*Isochrysis galbana*	50–56	12–15	10–20	[161]
*Nannochloropsis oceanica*	40–45	12–15	25–30	[158]
*Nannochloropsis granulata*	30–40	20–25	30–40	[163]
*Nostoc sphaeroides*	50.8	15.1	14.5	[158]
*Phaeodactylum tricornutum*	38.8	20.5	11	[158]
*Synechococcus spp*.	63	11	15	[164]
*Spirulina maxima*	60–70	6–7	13–16	[161]
*Tetraselmis chuii*	46.3	12.3	25	[163]

**Table 7:** Use of SCP as feed ingredients in different aquaculture species.

Aquaculture species	Species	Inclusion (%)	Effects	References
Pacific white shrimp (*L. vannamei*)	*Tetraselmis suecica*	0.25, 0.50, 0.75	*L. vannamei* showed the highest weight gain and SGR when fed a diet supplemented with 0.75% dried *T. suecica*.	[186]
Shrimp (*P. monodon*)	*Schizochytrium limacinum*	0.75, 1.5	0.75% inclusion level in the diet improved the immune response and intestinal health of *P. monodon*.	[187]
Gibel carp (*C. gibelio*)	*Scenedesmus ovalternus*	0, 4	4% inclusion level enhanced the resistance of gibel carp against *A. hydrophila*.	[188]
Giant freshwater prawn (*M. rosenbergii*)	*Saccharomyces cerevisiae*	0–60	Up to 60% of FM protein could be replaced with *S. cerevisiae* in giant freshwater prawn diets.	[189]
Nile tilapia (*O. niloticus*)	*Rhodotorula mucilaginosa*	0–1	Including *R. mucilaginosa* in the diet can improve growth performance, nutrient profile, immune function, and antioxidant capacity in Nile tilapia.	[190]
Pacific white shrimp (*L. vannamei*)	*S. cerevisiae*	1	1% inclusion of can enhance growth performance, boost innate immunity, and improve resistance in *L. vannamei*.	[191]
Pacific white shrimp (*L. vannamei*)	*Schizochytrium *sp.	0, 2, 4, 6	2% and 4% inclusion of Schizochytrium sp. improved the specific growth rate of *L. vannamei.*	[184]
Giant freshwater prawn (*M. rosenbergii*)	*C. vulgaris*	0, 2, 4, 6, 8	Enhanced the growth rate and survival of post larvae compared to the FM-based control diet. Additionally, it increased the total hemoglobin count and improved post-larval survival against *A. hydrophila* infection compared to other diets.	[192]
Nile tilapia (*O. niloticus*)	*Schizochytrium* sp.	25, 50, 75, 100	Improved weight gain, FCR, and PER were observed, particularly when fish oil was fully replaced with Schizochytrium at 100%	[193]
Giant freshwater prawn (*M. rosenbergii*)	*Arthrospira platensis*	25, 50, 75, 100	Replacement of fishmeal at 50% significantly improved growth, feed efficiency, and nutrient composition in *M. rosenbergii*. Digestive enzyme activity also increased, indicating 50% replacement as the optimal level.	[194]
Gourami (Trichopodus trichopterus)	*S. platensis*	2.5, 5, 10, 20	8.1–9.6% inclusion showed better growth performance	[195]
Pacific white shrimp (*L. vannamei*)	Biofloc meal	0, 7.5, 15, 30	30% inclusion showed significantly better performance in weight gain, final weight, SGR, and PER than all other treatments	[196]
Rainbow trout (*O. mykiss*)	*A. platensis*	0, 2.5, 5, 7.5, 10	10% inclusion level significantly increased RBC and WBC counts, along with total protein and albumin levels, in Rainbow trout	[197]
Pacific white shrimp (*L. vannamei*)	*Dunaliella *sp.	1, 2	The survival rate of *L. vannamei* increased with the inclusion of *Dunaliella *sp. in the diets	[198]
Nile tilapia (*O. niloticus*)	*A. platensis*	0.5–2	Significantly enhanced the health status of fish and boosted antioxidant function	[199]
Rainbow trout (*O. mykiss*)	*Spirulina* sp.	0, 2.5, 5, 7.5, 10	7.5% and 10% inclusion of Spirulina sp. significantly enhanced weight gain in rainbow trout	[200]
Common carp (*C. carpio*)	*Nanofrustulum* sp.	5–10	5%–10% inclusion of *Nanofrustulum* sp. in the diet of *C. carpio* resulted in superior growth and feed intake compared to FM	[180]
Gilthead sea bream (*S. aurata*)	*Nannochloropsis gaditana*	0.5–1	0.5%–1% inclusion of *N. gaditana* enhanced the immune system of *S. aurata*	[201]
Golden barb (*P. gelius*)	*S. platensis*	0, 5, 10, 20	20% inclusion of *S. platensis* significantly improved the growth rates of golden barb	[202]
Atlantic cod (Gadus morhua)	*Nannochloropsis* sp. and Isochrysis sp.	0, 15, 30	Fish fed a diet with 15% FM replacement showed improved feed intake over time	[203]
Shrimp (*P. monodon*)	*Dunaliella salina*	0.5, 1, 2	Enhanced immunity, improved antioxidant activity, and increased survival rates were observed in *P. monodon*, indicating its potential as a prophylactic agent against WSSV infection in shrimp	[204]

## Data Availability

The data will be made available upon request.
